# Laparoscopic lumbar artery ligation of type II endoleaks following endovascular aneurysm repair: A case report

**DOI:** 10.1097/MD.0000000000025732

**Published:** 2021-05-07

**Authors:** Byeoung Hoon Chung, Hee Chul Yu, Jae Do Yang, Mi Rin Lee, Min Ro Lee, Hong Pil Hwang

**Affiliations:** a Department of Surgery, Jeonbuk National University Medical School; b Research Institute of Clinical Medicine of Jeonbuk National University-Biomedical Research Institute of Jeonbuk University Hospital, Jeonju, South Korea.

**Keywords:** abdominal aortic aneurysms, endovascular aneurysm repair, laparoscopy, lumbar artery ligation, type II endoleaks

## Abstract

**Introduction:**

Although the clinical significance of type II endoleaks remain controversial, management strategies continue to expand. The laparoscopic approach is a minimally invasive method for persistent type II endoleak repair after endovascular aneurysm repair.

**Patient concerns:**

A 70 - year - old male patient with a history of endovascular aneurysm repair with left internal iliac artery embolization presented with persistent type II endoleak from the lumbar arteries 2 years ago. The aneurysm sac size had increased more than 10 mm during follow up period.

**Diagnosis:**

Persistent type II endoleak after endovascular aneurysm repair.

**Interventions:**

Transarterial embolization was attempted and failed. A minimally invasive laparoscopic lumbar artery ligation was then utilized.

**Outcomes:**

The patient was discharged without any complications after surgery. Follow-up computed tomography angiography has shown the complete disappearance of the type II endoleaks.

**Conclusions:**

Laparoscopic lumbar artery ligation may be a safe and effective alternative treatment for type II endoleaks, especially in high resource settings.

## 1 Introduction

Since the 1900 s, endovascular aneurysm repair (EVAR) has been increasingly used for the management of abdominal aortic aneurysms. Compared to open surgical repair, the perioperative mortality of EVAR is lower. However, late complications such as endoleaks have been observed, which require lifelong follow-up.^[1–5]^ White et al. first proposed the term ‘endoleak’ in 1996^[6]^ and subdivided it into four classed in 1998.^[7]^ In particular, type II endoleak is a result of the retrograde flow of blood from collateral arteries, which may cause sac expansion and rupture over time.^[8,9]^ Recent guidelines recommend that type II endoleak management is warranted in the setting of aneurysm sac growth.^[10]^ Endovascular coil embolization is usually considered as a first-line treatment for type II endoleaks.^[11]^ However, the rate of persistent or recurrence is still high, especially, in cases of lumbar artery embolization (low mid-term success rate).^[12]^

Laparoscopic repair of type II endoleak is a minimally invasive alternative treatment modality to coil embolization that enables a definite ligation of the aortic collaterals for inflow and outflow of type II endoleaks.^[13]^ However, due to the highly inflamed surrounding aortic tissue after EVAR, this method has been technically challenging.^[14]^

Here, we report the use of laparoscopic transabdominal lumbar artery ligation in a patient with persistent type II endoleaks after EVAR.

### 1.1 Ethical approval

This case report was approved by the Jeonbuk National University Hospital Institutional Review Borad (2021-01-029), and the written informed consent was obtained from the patient for publication of this case report.

## 2 Case report

### 2.1 Patient characteristics

**Figure 1:**
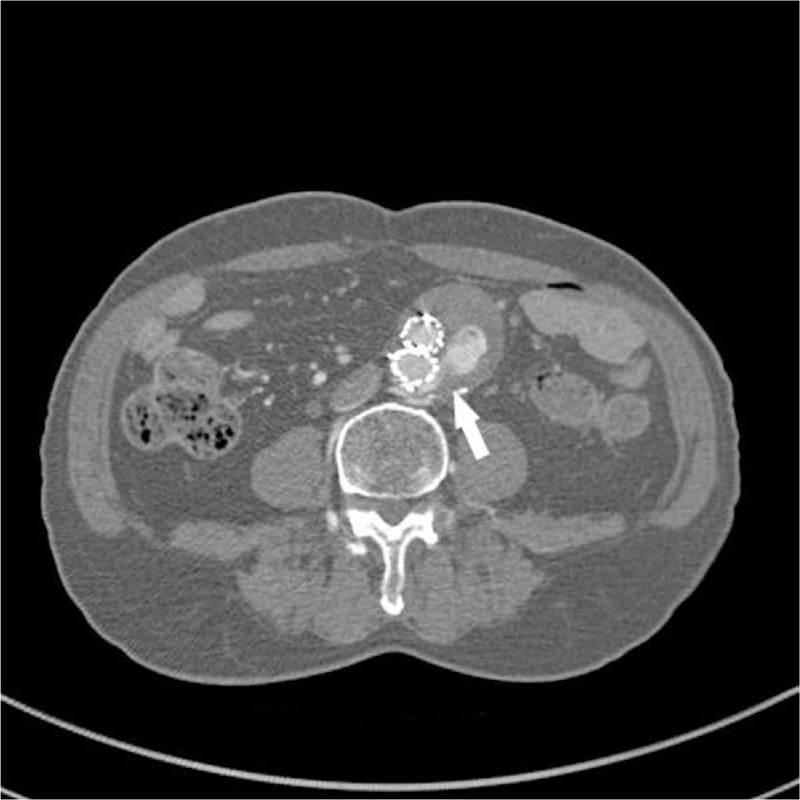
Axial view of two-year follow-up computed tomography angiography (CTA) taken 2 years after EVAR shows massive type II endoleak from lumbar arteries (arrow) with over 10 mm increase in sac diameter.

A 79 - year - old man underwent EVAR with left internal iliac artery embolization for a 53 mm abdominal aortic aneurysms in May 2018. A bifurcated Endurant II (Medtronic, CA, USA) was implanted and one Amplatzer vascular plug II (12 mm) was used to embolize the left internal iliac artery. On the fourth post-operative day, he was discharged without any complications. Two year later, computed tomography angiography revealed the presence of type II endoleaks from lumbar arteries with about 10 mm increase in aortic sac diameter (Fig. 1). Micro-coil embolization was attempted through the transarterial and translumbar approaches. However, these, failed because the vessel was not selected.

### 2.2 Operative techniques

**Figure 2:**
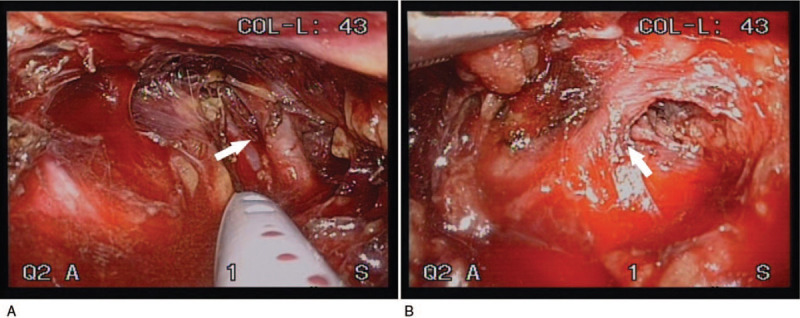
A, Intraoperative view reveals the left lumbar artery (arrow) identified through a transperitoneal approach. B, Right lumbar artery (arrow) dissection between the left side of the inferior vena cava and the right side of the aorta.

No other sources of the endoleaks were identified through imaging investigation. Hence, we decided transperitoneal laparoscopic lumbar artery ligation. Because the vascular surgeon lacked experience with the laparoscopic approach, an experienced colorectal specialist with extensive experience in lumbar artery dissection participated in this surgery. The surgery was performed using 1 port on umbilicus and 2 ports on each side of the patient. The left side of the aorta was exposed by sharp dissection of the sigmoid colon mesentery. The dorsal surface of the aorta was subsequently dissected until the left lumbar artery was identified. The left lumbar artery was then ligated using laparoscopic clips and a hemostatic energy device (Ligasure). The right lumbar artery was identified by a sharp dissection between the left side of the inferior vena cava and the right side of the aorta. The right lumbar artery was ligated through the same method used for the left lumbar artery (Fig. 2). The total operative time was 109 minutes.

### 2.3 Outcomes

**Figure 3:**
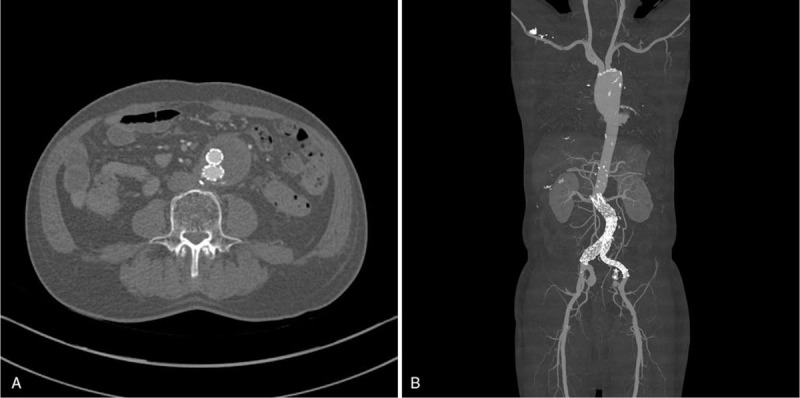
Post-operative CTA shows the complete elimination of type II endoleak. A, Axial view of CTA, B, Reconstruction view of CTA.

The follow-up period lasted 12 months; the hospitalization lasted 8 nights. Post-operative computed tomography angiography (post-operative day 4th) has shown the complete elimination of type II endoleak (Fig. 3). On the sixth and seventh post-operative days, the drain tube and stitches were removed, respectively. On eighth post-operative day, the patient was discharged without any complications.

## 3 Discussion

EVAR has been proven to reduce 30-day mortality up to 70% compared to open surgical repair.^[15]^ This initial benefit is maintained for up to two years, followed by relatively similar mortality due to the accumulation of EVAR-related complications.^[16,17]^ Given the high frequency of type II endoleaks following EVAR (10%-25%), subsequent management also causes continuous problems for interventional radiologist and vascular surgeons.^[18]^ Type I and III endoleaks carry a significant risk of aneurysmal rupture, however, opinions regarding sac expansion to the type II endoleaks differ considerably.^[19]^ It has been suggested that the risk of rupture may be high in the presence of type II endoleaks with significant sac enlargement.

Recent literature has suggested that conservative management with closed surveillance should be used to retain treatment for cases with (1) a more than 5 mm sac expansion over six months or (2) over 10 mm increase from the previous sac diameter.^[8]^ Other systematic reviews have also shown that 35% of the indications for treating type II endoleaks are previous failed endovascular interventions, and alternative treatments, such as laparoscopic arterial ligation, are necessary.^[20]^ Given the limited large prospective or randomized trials for type II endoleaks, the value of patient and clinician preferences in determining intervention or surgical treatment is still high.^[21]^

Until now, less invasive treatment options have emerged as viable alternatives, including endovascular coiling, glue embolization, sac embolization, graft explantation, and laparoscopic arterial ligation.^[18]^ Among these approaches, transarterial endovascular coiling is the most widely used. Despite initial data suggesting that this approach was robust to type 2 endoleak control, recent evidence has been less favorable to most patients during the 5 years of sustained sac expansions that require secondary procedures.^[18]^ This may be due to the intrinsic unique ability of the arterial supply to recanalize over time.^[22]^ In this regards, the laparoscopic approach has continued to evolve in an attempt to provide safe and reliable treatment.

The European Society of Vascular Surgery guidelines recommends that surgical treatment should be reserved for cases with failed endovascular intervention.^[23]^ However, high technical success rates (90%) and low 30-day mortality rates (0 to 1.5%) have been presented in recent systematic reviews of surgical approaches.^[20,24]^ In addition, it spares patients from exposure to harmful radiation typically used in endovascular interventions.^[8,20]^ Therefore, laparoscopic ligation may be considered as a safe and feasible treatment modality.

## 4 Conclusions

Despite the consistent updated form meta-analysis, systematic review, and clinical guidelines for type II endoleaks after EVAR, many controversial clinical questions remain. Furthermore, recommendations from European Society of Vascular Surgery regarding the management if type II endoleaks are still weak. This paper highlights that for case where endovascular interventions fail and additional options are not possible. Vascular surgeons always need to be alert to changes and get the up-to-date information. If there is no access vessel for intervention or if the procedure fails, laparoscopic lumbar artery ligation can be effective treatment modality for type II endoleaks after EVAR.

## Author contributions

**Conceptualization**: Byeoung Hoon Chung, Hee Chul Yu, Hong Pil Hwang

**Data curation:** Byeoung Hoon Chung, Mi Rin Lee.

**Investigation**: Byeoung Hoon Chung, Mi Rin Lee, Hong Pil Hwang.

**Methodology:** Min Ro Lee, Hong Pil Hwang.

**Resources**: Min Ro Lee, Hong Pil Hwang

**Supervision:** Hee Chul Yu, Hong Pil Hwang.

**Writing – original draft**: Byeoung Hoon Chung, Mi Rin Lee

**Writing – review & editing:** Byeoung Hoon Chung, Jae Do Yang, Hong Pil Hwang, Hee Chul Yu, Min Ro Lee.
